# Supplementary figures and images for: Intense pulsed light plus meibomian gland expression versus intense pulsed light alone for meibomian gland dysfunction: A randomized crossover study
Source: PLoS One. 2021 Mar 4;16(3):e0246245. doi: 10.1371/journal.pone.0246245 (PMC7932142; doi:10.1371/journal.pone.0246245)

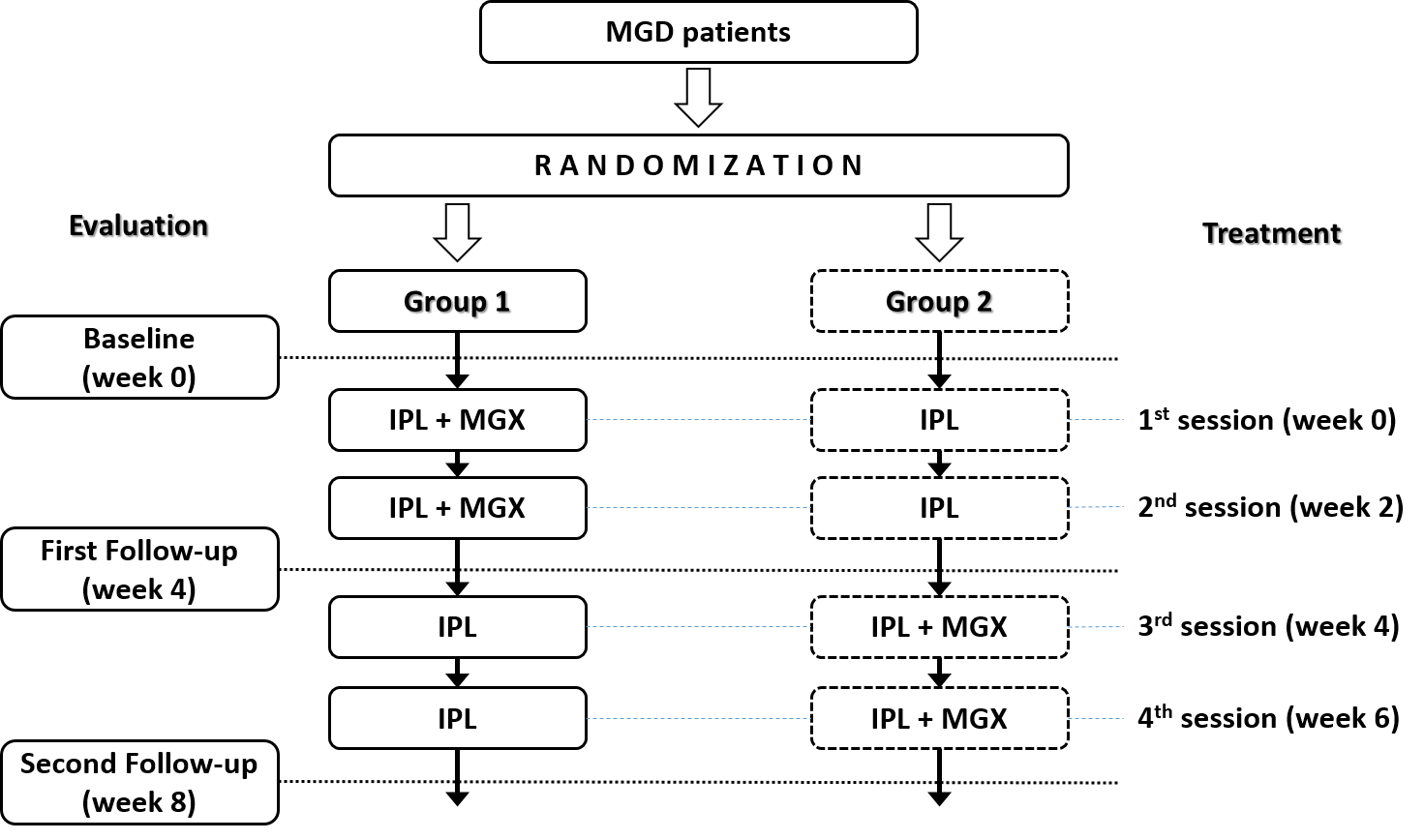

Supplement: S1 Fig — (TIF) [file pone.0246245.s002.tif]
