# Supplementary material for: Intense pulsed light plus meibomian gland expression versus intense pulsed light alone for meibomian gland dysfunction: A randomized crossover study
Source: PLoS One. 2021 Mar 4;16(3):e0246245. doi: 10.1371/journal.pone.0246245 (PMC7932142; doi:10.1371/journal.pone.0246245)
Supplement: S1 File — (DOC) [file pone.0246245.s003.doc]

**
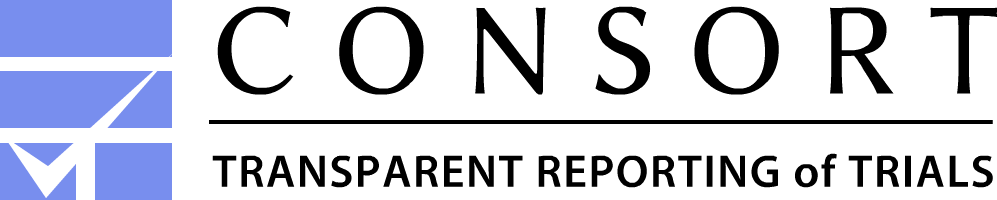
**

**CONSORT 2010 Flow Diagram**

**Allocation**

**Analysis**

**Follow-Up**

**Enrollment**

Assessed for eligibility (n = 85)

Excluded (n=13)

♦  Declined to participate (n=13)

Analysed (n = 33)

Lost to follow-up (n = 3)

Allocated to intervention (n = 36)

♦ Received allocated intervention (n = 36)

Lost to follow-up (give reasons) (n = 9)

Allocated to intervention (n = 36)

♦ Received allocated intervention (n = 36)

Analysed (n = 27)

Randomized (n = 72)
