# Supplementary material for: Intense pulsed light plus meibomian gland expression versus intense pulsed light alone for meibomian gland dysfunction: A randomized crossover study
Source: PLoS One. 2021 Mar 4;16(3):e0246245. doi: 10.1371/journal.pone.0246245 (PMC7932142; doi:10.1371/journal.pone.0246245)
Supplement: S2 File — (PDF) [file pone.0246245.s004.pdf]

Intense Pulsed Light plus Meibomian Gland Expression versus Intense Pulsed Light alone for Meibomian Gland Dysfunction: A Randomized Crossover Study

By Dong Hui Lim

WORD COUNT 5533
TIME SUBMITTED 16-NOV-2020 01:23PM
PAPER ID 65162270

Intense Pulsed Light plus Meibomian Gland Expression versus Intense Pulsed Light alone for Meibomian Gland Dysfunction: A Crossover Study

Kyoung Yoon Shin, MD[1*], Dong Hui Lim, MD, PhD[1*], Chan Hee Moon, MD[2], Byung Jin Kim, MD[2†], Tae-Young Chung, MD, PhD[1†]

[1]Department of Ophthalmology, Samsung Medical Center, Sungkyunkwan University School of Medicine, Seoul, Republic of Korea

[2]Department of Ophthalmology, Samsung Eye Clinic, Seoul, Republic of Korea

[*]These authors contributed equally as first authors.

[†]These authors contributed equally as corresponding authors.

Correspondence

Tae-Young Chung, MD, PhD

Department of Ophthalmology, Samsung Medical Center, Sungkyunkwan University School of Medicine, 81 Irwon-ro, Gangnam-gu, Seoul 06351, Republic of Korea

Tel: 82-2-3410-3548; Fax: 82-2-3410-0074; E-mail: tychung@skku.edu

Byung Jin Kim, MD

Department of Ophthalmology, Samsung Eye Clinic, 114, Olympic-ro, Songpa-gu, Seoul 05558, Republic of Korea

Tel: 82-2-424-7523; Fax: 82-2-3410-0074; E-mail: feeloph@naver.com

Running Head: IPL plus MGX versus IPL alone for MGD

Keywords: meibomian gland dysfunction, meibomian gland expression, intense pulsed light

ABSTRACT

Purpose: To investigate the comparative efficacy of intense pulsed light (IPL) therapy alone with that of IPL plus meibomian gland expression (MGX) for meibomian gland dysfunction (MGD).

Methods: This is a prospective randomized crossover clinical trial. Sixty patients were enrolled and randomly assigned to two groups. All of patients underwent four treatment sessions in total, which were two weeks apart. Group 1 underwent two sessions of IPL therapy with MGX, as well as two sessions of IPL alone. Group 2 received two sessions of IPL therapy alone, and two sessions of IPL therapy with MGX. The following parameters were measured at baseline (BL), 2 weeks after the second treatment session (FU1), and 2 weeks after the fourth treatment session (FU2): tearfilm break-up time (BUT), Oxford grade for corneal staining, meibomian gland expressibility (MGE), meibum quality (MQ), and ocular surface disease index (OSDI). The separate effect of MGX on improvement of MGD parameters was evaluated using generalized estimating equation (GEE).

Results: The mean age of the participants was 57.52 ± 10.50 years. The BUT, Oxford grade, MGE, MQ, and OSDI of both groups improved significantly (from baseline) by the end of four treatment sessions (FU2 compared to BL; all p-values <0.05). The MGE and MQ significantly improved after the first and second treatment sessions (FU1 compare to BL; all p-values < 0.001). However, the improvement was not statistically significant after the third and fourth treatment sessions (FU2 compared to FU1; p-value of 0.388 for MGE and 0.645 for MQ in group 1, 0.333 for MGE and 0.333 for MQ in group 2). The IPL plus MGX therapy produced greater improvements in the BUT scores than did IPL therapy alone (p=0.003 by GEE). In contrast, the Oxford grade, MGE, MQ, and OSDI were not influenced by the addition of MGX to IPL (p=0.642, 0.663, 0.731, and 0.840, respectively by GEE).

Conclusion: IPL therapy effectively improves the subjective symptoms and objective ocular findings of MGD. MGX enhanced the improvement of BUT driven by IPL therapy. The meibomian gland function (MGE and MQ) recovers faster in response to IPL therapy than did the other parameters.

INTRODUCTION

Meibomian gland dysfunction (MGD) is a chronic, diffuse abnormality of the meibomian glands which results in qualitative or quantitative changes in the secretion of meibom. MGD affects the tear film and causes eye irritation/inflammation, and ocular surface disease.[1] MGD is one of the most common disorders encountered in ophthalmology clinics and is considered to be a major cause of dry eye syndrome.[2,3] Because of this, it can be considered a public health problem, affecting up to 20% of the population in Europe and up to 60% in Asia.[1,4]

The current methods of treating MGD involve heat in the form of warm compresses, a heated pad or goggles,[5-8] self-administered lid massage, and manual expression.[9,10] Several novel methods have also been investigated. The positive ophthalmic effects of intense pulsed light (IPL) on patients undergoing treatment for facial rosacea was discovered,[11] and IPL has gained clinicians attention as a treatment for the MGD.

IPL therapy is widely used in the cosmetic industry and for removal of hypertrichosis, benign cavernous hemangiomas, benign venous malformations, telangiectasias, port-wine stains, and pigmented lesions.[12] After IPL was recognized to be beneficial for MGD, several additional studies using IPL were performed for MGD treatment.[13-21] Most of the IPL treatments were performed with meibomian gland expression (MGX). However, Craig et al.[13] and Jiang et al.[19] reported that IPL treatment alone can also improve the symptoms and signs of MGD. However, there has been no comparative study of IPL therapy and combination therapy (of IPL and MGX).

Therefore, in the current study, we investigated the treatment efficacy of combined therapy with MGX and IPL for MGD. This study is the first to compare IPL treatment alone with that of IPL and MGX.

METHODS

Setting

This is a prospective randomized clinical trial with a crossover design that compares the clinical outcomes of IPL alone with those of MGX plus IPL in MGD treatment. The study was approved by the Institutional Review Board (IRB) of Samsung Medical Center (IRB no. 2019-04-066) and adheres to the tenets of the Declaration of Helsinki. Written informed consent was obtained from all participants. The board approved the study on April 15th, 2019 and completed the study on April 8th, 2020. The study is registered at ClinicalTrials.gov (identifier, NCT03950115; date of registration, 15/05/2019). The study was initiated after the approval of IRB, but the posting of this study to ClinicalTrials.gov was after the initial enrollment due to the delay in online PRS (protocol registration and results system) review process. The authors confirm that all ongoing and related trials for this drug/intervention are registered.

Participants and design

Patients diagnosed with MGD in their both eyes between April 18th, 2019 and October 28th, 2019 were enrolled in the study and treated by two ophthalmologists (T-Y.C. and B.J.K.) at Samsung Medical Center and Samsung Eye Clinic. The diagnosis of MGD was according to Japanese MGD diagnostic criteria; MGD was considered to be present when all of the following three signs/findings are present: (1) chronic ocular discomfort, (2) anatomic abnormalities around the meibomian gland orifices, and (3) obstruction of the meibomian glands.[22] Prior to enrollment, participants were screened for general health and current/recent use of medications. Participants were excluded if they had a medical condition (including pregnancy, breastfeeding, lupus, and any major uncontrolled health problem) in which IPL is contraindicated. Participants who wear contact lens or punctal plugs, had recent ocular surgery, recent thermal treatment for dry eye disease (e.g., LipiFlow), or recent meibomian gland expression were also excluded. The enrolled patients were allocated randomly with equal probability into two groups by independent clinical trial consultants. All patients underwent four treatment sessions two weeks apart in both eyes. Group 1 underwent IPL therapy with MGX at the first and second treatment sessions and IPL therapy alone at the third and fourth treatment sessions. Group 2 received IPL therapy alone at the first and second treatment sessions and IPL therapy with MGX at the third and fourth treatment sessions. The overall study design is summarized in Figure 1.

Intervention

IPL therapy was performed with the M22® (Lumenis, Dreieich, Germany) and administered to the skin below the lower eyelid. Before treatment, the eyes were protected with opaque goggles. Ultrasound gel was

applied to the patient's face from tragus to tragus including the nose to conduct the light, help spread the energy evenly, and provide a degree of protection.[11] The intensity of IPL treatment ranged from 9.8J/cm2 to 13J/cm2 according to Fitzpatrick Skin Type Grade.[13] MGX was performed immediately after IPL treatment on both upper and lower eyelids of each eye. To minimize pain during this procedure, the eye was numbed with a solution of proparacaine HCl 0.5% (Alcaine; Alcon Laboratories, Fort Worth, TX). MGX was performed by squeezing the meibomian glands with meibomian gland expressor forceps or with two Q-tips positioned on either side of the meibomian glands.

Outcomes

Patients were evaluated immediately before the first treatment session (or the baseline [BL]), immediately before the third treatment session (first follow-up [FU1]), and two weeks after the fourth treatment session (second follow-up [FU2]). From BL to FU2, each patient was treated and followed for a total of eight weeks.

The meibograde was measured at BL using the Keratograph ® 5M (Oculus, Wetzlar, Germany) and graded using the Pult H method: 0 (meibomian gland area of loss = 0%), 1 (area of loss < 25%), 2 (area of loss 25-50%), 3 (area of loss 51-75%), and 4 (area of loss >75%).[23] The severity of dry eye symptoms was evaluated using the Ocular Surface Disease Index (OSDI).[24] The severity of meibomian gland function was evaluated using the meibomian gland expressibility (MGE) score and the meibum quality (MQ) score. The MGE was assessed on a scale of 0 to 3 in five glands on the central lower lid and was scaled according to number of expressible glands as follows: 0 (all glands), 1 (three to four glands), 2 (one to two glands), and 3 (no glands).[25] Secretion quality was divided into the following four degrees: 0 (clear), 1 (cloudy), 2 (granular), and 3 (toothpaste).[26] We measured non-invasive tear break-up time (BUT) using Keratograph® 5M and fluorescein corneal staining grade to evaluate the ocular surface. Fluorescein corneal staining was enhanced by a yellow filter and graded using the Oxford Score (0 to 5 for the total cornea).[27]

Sample size calculation, randomization, and masking

A total sample size of is 72 with an equal number in each sequence (i.e., a total of 144 repeated measurements) is required to infer that the mean difference in score improvement between two treatments ('IPL only' vs 'IPL plus MGX') is not equal to 0, when the medium effect size of 0.5 is considered, the significance level is 0.05, the power of 80% is expected for a two-sided t-test in the repeated ANOVA with a 2-period by 2-treatment cross-over design,[28] and the drop-out rate is 10%. 36 subjects were assigned to each treatment sequence, but 3 subjects in group 1 and 9 subjects in group 2 were dropped-out during the follow-up. The drop-out rate was more than anticipated, but this reduced sample size of 60 subjects may not lead to great reduction on the

expected power of 80% for the following reasons. First, we used the GEE method that usually has higher power than LMM methods, including the repeated ANOVA.[29] Second, the data were collected from both eyes for each subject, and hence a total of 240 repeated measurements were used for analysis. This would help power increment to a certain degree.

The randomization process was implemented by independent clinical trial statisticians. Patients were randomized in 1:1 ratio using block randomization method with permutated blocks of 4 or 6 in size based on pre-allocated codes placed in sealed opaque envelopes that were opened during the randomization step. Due to the nature of the intervention, participants, healthcare professionals and researchers could not be blinded to group allocation. Only trial statisticians were masked to allocation.

Statistical analyses

Clinical features of both eyes of the participants were analyzed. The clinical parameters of both groups were compared at each point of the evaluation (BL, FU1, FU2) using the Wilcoxon rank-sum test. Analysis of the improvement after therapy was performed using Wilcoxon signed-rank test, which compared the BUT, Oxford grade, MGE, MQ, and OSDI scores at BL, FU1, and FU2. To separately evaluate the effect of MGX on score improvement, the generalized estimating equation (GEE) method was employed because it is known to be robust against the incorrect specification of the correlation structure among repeated measurements and hence produces consistent estimates, compared to the linear mixed effect model (LMM) method.[30,31] In the analysis of repeated measurements from a cross-over design, GEE methods usually showed better performance than LMM methods.[29] For the GEE model, dependent variables were defined as score changes from baseline to follow-up evaluations. First, univariable analysis was performed with age, sex, and baseline parameters (meibograde, BUT, Oxford grade, MGE, MQ, and OSDI) as confounders. In addition, any parameters with a p-value <0.1 on univariable analysis were adjusted at the final GEE analysis model for MGX effect. All statistical analyses were performed using Statistical Analysis System software version 9.4 (SAS Inc. Cary. NC).

RESULTS

The baseline demographics are shown in Table 1. Sixty participants (19 males and 41 females) finished all four treatment sessions and underwent final evaluation. Group 1 comprised 66 eyes from 33 subjects and Group 2 included 54 eyes from 27 subjects. The mean participant age was 57.52 ± 10.50 years (range, 32–78 years). The baseline meibograde was 2.19 ± 0.98, BUT was 4.49 ± 1.32, Oxford corneal staining grade was 1.46 ± 0.62, MGE score was 1.95 ± 0.85, MQ score was 2.09 ± 0.56, and OSDI score was 61.41 ± 20.85. Overall, the participants had severe dry eye symptoms and moderate to advanced MGD at baseline. There were no statistically significant differences between the two groups regarding baseline meibograde, BUT, Oxford grade, MGE, MQ, and OSDI.

Table 1. Baseline characteristics of the study subjects.

	**Total**	**Group 1**	**Group 2**	**p-value***
**Number of patients (eyes)**	60 (120)	33 (66)	27 (54)	
**Age**	57.52 ± 10.50	58.00 ± 10.73	56.93 ± 10.38	0.845
**Sex (male:female)**	19:41	10:23	9:18	1.000
**Meibograde**	2.04 ± 1.10	2.19 ± 1.11	1.87 ± 1.06	0.165
**BUT**	4.39 ± 1.50	4.02 ± 1.75	4.60 ± 1.32	0.397
**Oxford Grade**	1.46 ± 0.62	1.42 ± 0.59	1.50 ± 0.67	0.608
**MGE**	1.95 ± 0.85	1.97 ± 0.77	1.93 ± 0.95	0.837
**MQ**	2.09 ± 0.56	2.08 ± 0.61	2.11 ± 0.50	0.864
**OSDI**	61.41 ± 20.85	58.86 ± 19.88	64.92 ± 21.88	0.067

Numerical continuous parameters were described as means ± standard deviations, and categorical parameters were described as total numbers.

BUT=tear film break up time; MGE=meibomian gland expressibility score; MQ = meibum quality score; OSDI=Ocular Surface Disease Index

*p-values were obtained using the Wilcoxon rank-sum test for continuous data and the Fisher's exact test for categorical data

Figure 2 demonstrates improvement in MGD indices after treatment sessions. Group 1 had a better BUT score on the first follow-up compared to that of group 2 (p=0.049). However, this difference was not observed at the second follow-up. The Oxford grade, MGE, MQ, and OSDI did not differ between groups 1 and 2 at any point in the evaluation. Compared to other parameters, MGE and MQ tended to respond faster to treatment than the other parameters. Therefore, most of the improvement in MGE and MQ occurred between baseline and the first follow-up visit. Table 2 shows that the ocular surface health (BUT, Oxford grade), meibomian gland function

(MGE, MQ), and dry eye symptoms (OSDI) of both groups significantly improved by the end of the four treatment sessions (FU2 compared to BL). All parameters significantly improved at the first or second treatment session (FU1 compared to BL). However, only BUT, Oxford grade, and OSDI improved at the third and fourth treatment sessions in both groups (FU2 compared to FU1). There was no significant improvement in MGE or MQ between FU1 and FU2 in either group 1 or 2.

Figure 2. Improvement of tear film break up time (BUT), Oxford grade, meibomian gland expressibility (MGE), meibum quality (MQ), and Ocular Surface Disease Index (OSDI) after treatment. Group 1 received IPL plus MGX at the first and second treatment sessions and IPL alone at the third and fourth treatment sessions. Group 2 received IPL alone at the first and second sessions and IPL plus MGX at the third and fourth sessions. At baseline (BL), the two groups were comparable in every parameter. However, at the first follow-up (FU1), group 1 showed improved BUT (*). At the second follow-up (FU2), none of the parameters differed between group 1 and group 2.

Table 2. Meibomian gland dysfunction scores before and after treatment.

	**BL**	**FU1**	**FU2**	**p (BL–FU2)**	**p (BL–FU1)**	**p (FU1–FU2)**
**Group 1**						
**BUT**	4.02 ± 1.75	6.02 ± 2.32	7.95 ± 2.54	**0.004**	**0.023**	**0.002**
**Oxford Gr**	1.42 ± 0.59	0.72 ± 0.65	0.45 ± 0.60	**<0.001**	**<0.001**	**0.042**
**MGE**	1.97 ± 0.77	1.56 ± 0.69	1.42 ± 0.75	**0.001**	**<0.001**	0.388
**MQ**	2.08 ± 0.61	0.20 ± 0.09	0.17 ± 0.09	**<0.001**	**<0.001**	0.645
**OSDI**	58.86 ± 19.88	41.23 ± 26.46	27.75 ± 11.55	**<0.001**	**0.040**	**0.002**
**Group 2**						
**BUT**	4.60 ± 1.32	4.86 ± 2.11	7.41 ± 3.37	**0.005**	**0.029**	**0.007**
**Oxford Gr**	1.50 ± 0.67	0.80 ± 0.76	0.38 ± 0.49	**<0.001**	**<0.001**	**0.030**
**MGE**	1.93 ± 0.95	1.56 ± 0.79	1.50 ± 0.51	**0.032**	**<0.001**	0.333
**MQ**	2.11 ± 0.50	0.19 ± 0.10	0.19 ± 0.06	**<0.001**	**<0.001**	0.333
**OSDI**	64.92 ± 21.88	39.20 ± 15.42	26.55 ± 13.95	**<0.001**	**<0.001**	**<0.001**

BL=baseline; FU1 (first follow-up) = 2 weeks after the second treatment session; FU2 (second follow-up) = 2 weeks after the fourth treatment session; BUT=tear film break up time; MGE=Meibomian gland expressibility score; MQ = meibum quality score; OSDI=Ocular Surface Disease Index;
p-values were obtained using Wilcoxon signed-rank test

Addition of MGX to IPL led to score improvement only in BUT (p=0.003) while no improvement was

observed in Oxford grade, MGE, MQ, and OSDI (Table 3). That is, the combination of MGX and IPL improved BUT score by 2.701 on average more than did IPL therapy alone.

Table 3. The separate influence of MGX on meibomian gland dysfunction improvement

	Beta	95% CI of Beta	p-value
**BUT**	2.701	(0.891,4.510)	0.003
**Oxford grade**	0.080	(-0.258, 0.419)	0.642
**MGE**	0.105	(-0.365, 0.574)	0.663
**MQ**	0.009	(-0.040,0.058)	0.731
**OSDI**	-1.352	(-14.464, 11.760)	0.840

MGX=meibomian gland expression; FU=follow-up; CI=confidence interval; BUT=tear film break up time; MGE=meibomian gland expressibility score; MQ = meibum quality score; OSDI=Ocular Surface Disease Index; The beta and p-values were calculated using a generalized estimating equation.

There were no significant adverse events during the study period.

DISCUSSION

This prospective crossover study demonstrates that IPL effectively improves subjective symptoms and objective ocular findings for MGD, and MGX along with IPL enhance improvement of BUT in MGD. Several previous studies found that IPL is effective in treatment of MGD. However, this study is the first to directly investigate the separate effect of combination therapy with MGX and IPL. Our study also revealed that meibomian gland function (MGE and MQ) recovers faster with IPL therapy than do the other MGD parameters. Our findings are important to establish optimal practice guidelines in reference to MGX and IPL in treatment of MGD.

MGD is a highly prevalent ocular surface disease and is one of the most common diseases encountered in an ophthalmology clinic. The conventional treatment for MGD remains transient, unsatisfactory, and not comprehensive. Therefore, there is a need for new therapeutic approaches to MGD. IPL treatment is a new treatment option for MGD patients and was incidentally found to be effective. The mechanism by which IPL is thought to be effective in MGD involves thermal heating of the glands, which melts the thickened meibum secretions and promotes gland dilation. [11] This dilation ultimately allows for effective clinical expression of the glands.[11] Other potential mechanisms for IPL to treat MGD include vascular thrombosis of abnormal blood vessels below the skin surrounding the eyes; activation of fibroblasts which leads to synthesis of new collagen fibers; reduction in bacterial and Demodex load on the eyelids; changes in levels of reactive oxygen species and inflammatory chemokines; and reduction in turnover of skin epithelial cells which cause obstruction of the meibomian glands.[14,18] Previous reports have shown significantly improved dry eye symptoms and meibomian gland function after combined therapy with IPL and MGX in participants with advanced MGD (that was non-responsive to LipiFlow treatment).[17] Since LipiFlow can also provide thermal gland heating and expression, these results suggest that IPL provides a therapeutic mechanism beyond that of thermal heating and expression alone.

The outcomes of IPL treatment of MGD in the current report are similar to recently published data. Craig et al.[13] found a benefit of IPL treatment without MGX in a prospective, double-masked, placebo-controlled, paired-eye study in a younger patient population (mean age 45 years) of 28 subjects. Subjects had improved lipid layer grade (p<0.001), noninvasive tear film BUT (p<0.001), and visual analog scale symptom score (p=0.015) in the study eye but showed no changes in tear meniscus height or tear evaporation rate with treatment. Similarly, Toyos et al.[11] reported a significant improvement in tear BUT in 87% of patients in a three-year retrospective review of 91 patients. In addition, 93% of the patients reported amelioration of symptoms

after treatment. Vora and Gupta[15] completed a retrospective review of patients with a diagnosis of evaporative dry eye disease who underwent three or more IPL treatments. These patients were evaluated at each visit for tear BUT, grade of eyelid and facial vascularity, eyelid margin edema, and meibom quality/flow and completed an OSDI questionnaire. From the first to last follow-up visit, there was a significant decrease in the clinical signs of MGD ($p<0.001$) and OSDI ($p<0.001$). There was also significant increase in oil flow score and tear BUT ($p<0.001$). Vegunta et al[17] reported a retrospective study of 81 patients with MGD and dry eye treated with serial IPL and MGX therapy. This group showed that the combination of IPL and MGX significantly improved dry eye symptoms (89% of subjects) and meibomian gland function (77% of subjects).[17]

Most IPL treatments for MGD were performed with MGX, while some other studies reported that IPL treatment alone can effectively improve the symptoms and signs of MGD. However, no prior study compared the treatment of MGD with combination therapy (with IPL and MPX) versus that of IPL alone. Therefore, our study is the first to demonstrate the separate effect of MGX upon IPL/MGX treatment for MGD. We found that BUT improvement is augmented by addition of MGX to IPL treatment. The effect of MGX toward other objective and subjective indices was not statistically significant.

There is a variety of methods for forceful expression of meibomian glands.[32-34] A limiting factor of all these methods, however, is associated pain that is only minimally relieved by topical anesthetics. Warm compresses and self-administered lid massage are frequently ineffective, and manual expression by a practitioner can be very painful for the patient.[35] The amount of pain increases rapidly as the force of expression exceeds 5 pounds per square inch (PSI)[35]. The usual maximal tolerable force is 15 PSI, which is frequently marginal or inadequate to express obstructive material.[35] To perform effective MGX, pain must be expected and tolerated. Therefore, before performing MGX for MGD, providers must consider the balance of BUT improvement with patient pain. The MGX should be deferred if a patient cannot tolerate the procedure due to pain.

There is no clinical guideline regarding optimal number of IPL treatments for MGD. In this study, BUT, MGE, MQ, Oxford grade, and OSDI value improved significantly from baseline to after four treatments (in both groups). This result is consistent with those of previous studies. Interestingly, meibomian gland function (MGE and MQ) responded rapidly to treatment and reached a plateau at FU1. There was no significant improvement in MGE or MQ between FU1 and FU2. In contrast, BUT and Oxford grade improved gradually from baseline to the last follow-up. Most previous studies of IPL in MGD only compared results from baseline and the last follow-up. Only one study reported serial changes in MGD symptoms/signs during IPL treatment sessions.[19] According to Jiang et al.,[19] MGD symptoms (including eyelid margin and meibomian gland assessments,

tearfilm BUT, conjunctival injection, and tear meniscus height), except corneal staining, significantly improved from baseline to treatment days D15, D45, and D75. However, they found no significant difference in symptoms or TBUT between D45 and D75. Based on these findings, we suggest that two sessions of IPL alone can effectively improve meibomian gland function (MGE and MQ), while four sessions are necessary for relieving corneal signs (BUT, Oxford grade) and subjective symptoms. Regardless, further studies are needed to define standards of IPL treatment for MGD.

Our study has several limitations. First, the last follow-up period was performed only 2 weeks after the end of all treatment sessions. This short follow-up period cannot predict the long-term outcomes of IPL and MGX treatments on MGD. A second limitation is that our study had a 2-week interval between treatments, which is different from the widely accepted protocol of a 3-week or longer interval. However, several studies have reported 2-week interval IPL treatment as effective in MGD and dry eye syndrome[36], and the disease begin to improve 2 weeks after IPL treatment[19,37]. Therefore, the investigators believe the protocol in the present study is also effective in treating MGD and is worth reporting. A third limitation is that the current study might not have a long enough washout period for a crossover study. Since IPL was performed in every participant at every session as a baseline treatment, and meibomian gland expression (MGX) is the cross-over treatment, the investigators based the washout period on treatment effect of MGX. However, the duration of effect of MGX has not been explored well in the literature. We thought that a 2-week interval was an acceptable washout period for the following two reasons. First, a previous study regarding treatment effect of MGX on MGD[38] used a treatment interval of only one week. In addition, most MGD patients who visited a clinic and received MGX require additional MGX at the next visit. Considering those points, we carefully assumed that that 2 weeks would be an acceptable interval for loss of MGX effect. Future studies addressing these issues including small sample size and much longer wash out period is warranted to better understand the benefits of the MGX combined with IPL for MGD. A fourth limitation is that the patients could not be blinded toward their treatment, which may have biased their subjective improvement. However, we recognize that the inability to blind is an inherent problem of IPL and MGX treatments. Graig et al.[13] previously discussed the inherent difficulties and limitations associated with IPL and MGX since it is almost impossible to blind patients to treatment. A fifth limitation is that we did not analyze lid margin telangiectasia, which is critical for diagnosis and efficacy monitoring of MGD. Further research is necessary to evaluate the role of MGX on lid margin telangiectasia of MGD patients. Despite these limitations, our study has several strengths. This is the first study to investigate the separate effect of combined IPL plus MGX for MGD. In addition, unlike most prior studies on IPL and MGX

for MGD, our study demonstrated serial improvements in MGD indices during subsequent treatments.

In conclusion, IPL is an effective treatment method for MGD. Addition of MGX to IPL augments the improvement in BUT provided by IPL alone. Only two sessions of IPL were needed to improve meibomian gland function (MGE and MQ). Four IPL sessions were necessary to improve corneal signs (BUT, Oxford grade) and subjective symptoms. We believe that our results provide valuable information for development of optimal practice guidelines regarding IPL and MGX in treatment of MGD.

ACKNOWLEDGEMENTS

We would like to thank Kyunga Kim, PhD and Min-Ji Kim, MS in Statistics and Data Center, Research Institute for Future Medicine at Samsung Medical Center for assistance with randomization and statistical analyses.

AUTHOR CONTRIBUTIONS

Tae-Young Chung and Byung Jin Kim designed the study. Kyoung Yoon Shin, Dong Hui Lim, Chan Hee Moon, Byung Jin Kim, and Tae-Young Chung analyzed the clinical data. Kyoung Yoon Shin, Dong Hui Lim, Byung Jin Kim, and Tae-Young Chung reviewed the design and results and wrote/reviewed the final paper. Kyoung Yoon Shin and Dong Hui Lim equally contributed to the manuscript as first authors. Byung Jin Kim and Tae-Young Chung contributed equally to the manuscript as corresponding authors. All authors read and approved the final manuscript.

FUNDING AND SUPPORT

None

CONFLICT OF INTEREST

No conflicting relationship exists for any author.

DATA AVAILABILITY

All relevant data are within the manuscript and its Supporting Information files.

REFERENCES

1. Nichols KK, Foulks GN, Bron AJ, et al. The international workshop on meibomian gland dysfunction: executive summary. *Invest Ophthalmol Vis Sci*. 2011;52(4):1922-1929.
2. Mathers WD. Ocular evaporation in meibomian gland dysfunction and dry eye. *Ophthalmology*. 1993;100(3):347-351.
3. Shimazaki J, Sakata M, Tsubota K. Ocular surface changes and discomfort in patients with meibomian gland dysfunction. *Arch Ophthalmol*. 1995;113(10):1266-1270.
4. Schaumberg DA, Nichols JJ, Papas EB, et al. The international workshop on meibomian gland dysfunction: report of the subcommittee on the epidemiology of, and associated risk factors for, MGD. *Invest Ophthalmol Vis Sci*. 2011;52(4):1994-2005.
5. Matsumoto Y, Dogru M, Goto E, et al. Efficacy of a new warm moist air device on tear functions of patients with simple meibomian gland dysfunction. *Cornea*. 2006;25(6):644-650.
6. Mori A, Oguchi Y, Goto E, et al. Efficacy and safety of infrared warming of the eyelids. *Cornea*. 1999;18(2):188-193.
7. Mitra M, Menon GJ, Casini A, et al. Tear film lipid layer thickness and ocular comfort after meibomian therapy via latent heat with a novel device in normal subjects. *Eye (Lond)*. 2005;19(6):657-660.
8. Nam SW, Lim DH, Hyun J, Chung T-Y. Effects and Prognostic Factors of Automated Thermodynamic System Treatment for Meibomian Gland Dysfunction. *J Korean Ophthalmol Soc*. 2016;57(5):724-733.
9. Craig JP, Blades K, Patel S. Tear lipid layer structure and stability following expression of the meibomian glands. *Ophthalmic Physiol Opt*. 1995;15(6):569-574.
10. Olson MC, Korb DR, Greiner JV. Increase in tear film lipid layer thickness following treatment with warm compresses in patients with meibomian gland dysfunction. *Eye Contact Lens*. 2003;29(2):96-99.
11. Toyos R, McGill W, Briscoe D. Intense pulsed light treatment for dry eye disease due to meibomian gland dysfunction; a 3-year retrospective study. *Photomed Laser Surg*. 2015;33(1):41-46.
12. Raulin C, Greve B, Grema H. IPL technology: a review. *Lasers Surg Med*. 2003;32(2):78-87.
13. Craig JP, Chen YH, Turnbull PR. Prospective trial of intense pulsed light for the treatment of meibomian gland dysfunction. *Invest Ophthalmol Vis Sci*. 2015;56(3):1965-1970.
14. Dell SJ, Gaster RN, Barbarino SC, Cunningham DN. Prospective evaluation of intense pulsed light and meibomian gland expression efficacy on relieving signs and symptoms of dry eye disease due to meibomian gland dysfunction. *Clin Ophthalmol*. 2017;11:817-827.
15. Gupta PK, Vora GK, Matossian C, et al. Outcomes of intense pulsed light therapy for treatment of evaporative dry eye disease. *Can J Ophthalmol*. 2016;51(4):249-253.
16. Vora GK, Gupta PK. Intense pulsed light therapy for the treatment of evaporative dry eye disease. *Curr Opin Ophthalmol*. 2015;26(4):314-318.

17. Vegunta S, Patel D, Shen JF. Combination Therapy of Intense Pulsed Light Therapy and Meibomian Gland Expression (IPL/MGX) Can Improve Dry Eye Symptoms and Meibomian Gland Function in Patients With Refractory Dry Eye: A Retrospective Analysis. *Cornea*. 2016;35(3):318-322.
18. Dell SJ. Intense pulsed light for evaporative dry eye disease. *Clin Ophthalmol*. 2017;11:1167-1173.
19. Jiang X, Lv H, Song H, et al. Evaluation of the Safety and Effectiveness of Intense Pulsed Light in the Treatment of Meibomian Gland Dysfunction. *J Ophthalmol*. 2016;2016:1910694.
20. Arita R, Mizoguchi T, Fukuoka S, Morishige N. Multicenter Study of Intense Pulsed Light Therapy for Patients With Refractory Meibomian Gland Dysfunction. *Cornea*. 2018;37(12):1566-1571.
21. Arita R, Fukuoka S, Morishige N. Therapeutic efficacy of intense pulsed light in patients with refractory meibomian gland dysfunction. *Ocul Surf*. 2019;17(1):104-110.
22. Amano S. Meibomian Gland Dysfunction: Recent Progress Worldwide and in Japan. *Invest Ophthalmol Vis Sci*. 2018;59(14):DES87-DES93.
23. Pult H, Nichols JJ. A review of meibography. *Optom Vis Sci*. 2012;89(5):E760-769.
24. Asiedu K, Kyei S, Mensah SN, et al. Ocular Surface Disease Index (OSDI) Versus the Standard Patient Evaluation of Eye Dryness (SPEED): A Study of a Nonclinical Sample. *Cornea*. 2016;35(2):175-180.
25. Tomlinson A, Bron AJ, Korb DR, et al. The international workshop on meibomian gland dysfunction: report of the diagnosis subcommittee. *Invest Ophthalmol Vis Sci*. 2011;52(4):2006-2049.
26. Arita R, Minoura I, Morishige N, et al. Development of Definitive and Reliable Grading Scales for Meibomian Gland Dysfunction. *Am J Ophthalmol*. 2016;169:125-137.
27. Bron AJ, Evans VE, Smith JA. Grading of corneal and conjunctival staining in the context of other dry eye tests. *Cornea*. 2003;22(7):640-650.
28. Julious SA. Sample sizes for clinical trials with normal data. *Stat Med*. 2004;23(12):1921-1986.
29.
30. LIANG K-Y, ZEGER SL. Longitudinal data analysis using generalized linear models. *Biometrika*. 1986;73(1):13-22.
31. Hubbard AE, Ahern J, Fleischer NL, et al. To GEE or not to GEE: comparing population average and mixed models for estimating the associations between neighborhood risk factors and health. *Epidemiology*. 2010;21(4):467-474.
32. Hom MM, Silverman MW. Displacement technique and meibomian gland expression. *J Am Optom Assoc*. 1987;58(3):223-226.
33. Korb DR, Greiner JV. Increase in tear film lipid layer thickness following treatment of meibomian gland dysfunction. *Adv Exp Med Biol*. 1994;350:293-298.
34. McCulley JP, Sciallis GF. Meibomian keratoconjunctivitis. *Am J Ophthalmol*. 1977;84(6):788-

793.

35. Korb DR, Blackie CA. Meibomian gland therapeutic expression: quantifying the applied pressure and the limitation of resulting pain. *Eye Contact Lens*. 2011;37(5):298-301.
36. Guilloto Caballero S, Garcia Madrona JL, Colmenero Reina E. Effect of pulsed laser light in patients with dry eye syndrome. *Arch Soc Esp Oftalmol*. 2017;92(11):509-515.
37. Piyacomn Y, Kasetsuwan N, Reinprayoon U, et al. Efficacy and Safety of Intense Pulsed Light in Patients With Meibomian Gland Dysfunction-A Randomized, Double-Masked, Sham-Controlled Clinical Trial. *Cornea*. 2019.
38. Lee H, Kim M, Park SY, et al. Mechanical meibomian gland squeezing combined with eyelid scrubs and warm compresses for the treatment of meibomian gland dysfunction. *Clin Exp Optom*. 2017;100(6):598-602.

Intense Pulsed Light plus Meibomian Gland Expression versus Intense Pulsed Light alone for Meibomian Gland Dysfunction: A Randomized Crossover Study

ORIGINALITY REPORT

11%

SIMILARITY INDEX

PRIMARY SOURCES

1	Vora, Gargi K., and Preeya K. Gupta. "Intense pulsed light therapy for the treatment of evaporative dry eye disease :", Current Opinion in Ophthalmology, 2015.<br>Crossref	63 words — 1%
2	www.clinicaltrials.gov<br>Internet	52 words — 1%
3	www.bktimes.net<br>Internet	48 words — 1%
4	www.tfosdewsreport.org<br>Internet	45 words — 1%
5	socosc.com<br>Internet	38 words — 1%
6	Sheng-nan Cheng, Fa-gang Jiang, Hua Chen, Hui Gao, Yu-kan Huang. "Intense Pulsed Light Therapy for Patients with Meibomian Gland Dysfunction and Ocular Demodex Infestation", Current Medical Science, 2019<br>Crossref	36 words — 1%
7	Seung Wan Nam, Dong Hui Lim, Kyu Yeon Cho, Hye Seung Kim, Kyunga Kim, Tae-Young Chung. "Risk factors of presenile nuclear cataract in health screening study", BMC Ophthalmology, 2018<br>Crossref	34 words — 1%
8	Sungsoon Hwang, Dong Hui Lim, Tae-Young Chung. "Prevalence	

and Incidence of Keratoconus in South Korea: A Nationwide Population-Based Study", American Journal of Ophthalmology, 2018
Crossref

34 words — 1 %

9 Preeya K. Gupta, Gargi K. Vora, Cynthia Matossian, Michelle Kim, Sandra Stinnett. "Outcomes of intense pulsed light therapy for treatment of evaporative dry eye disease", Canadian Journal of Ophthalmology / Journal Canadien d'Ophtalmologie, 2016
Crossref

31 words — 1%

10 www.science.gov
Internet

27 words — 1%

11 journals.lww.com
Internet

24 words — < 1%

12 synapse.koreamed.org
Internet

22 words — < 1%

13 arrow.tudublin.ie
Internet

19 words — < 1%

14 iovs.arvojournals.org
Internet

19 words — < 1%

15 Jeung Hui Pyo, Hyuk Lee, Byung-Hoon Min, Jun Haeng Lee et al. "Comparison of Long-Term Outcomes After Non-curative Endoscopic Resection in Older Patients with Early Gastric Cancer", Annals of Surgical Oncology, 2017
Crossref

15 words — < 1%

16 journals.plos.org
Internet

14 words — < 1%

17 Gyule Han, Jisang Han, Kyungdo Han, Jinyoung Youn, Tae-Young Chung, Dong Hui Lim. "Visual Acuity and Development of Parkinson's Disease: A Nationwide Cohort Study", Movement Disorders, 2020
Crossref

13 words — < 1%

18	www.thieme-connect.de<br>Internet	12 words — < 1%
19	mafiadoc.com<br>Internet	10 words — < 1%
20	effiloop.com<br>Internet	9 words — < 1%
21	www.lime.jp<br>Internet	9 words — < 1%
22	www.esw-vision.com<br>Internet	9 words — < 1%

EXCLUDE QUOTES	ON	EXCLUDE MATCHES	< 1%
EXCLUDE BIBLIOGRAPHY	ON		
