# Supplementary material for: Intense pulsed light plus meibomian gland expression versus intense pulsed light alone for meibomian gland dysfunction: A randomized crossover study
Source: PLoS One. 2021 Mar 4;16(3):e0246245. doi: 10.1371/journal.pone.0246245 (PMC7932142; doi:10.1371/journal.pone.0246245)
Supplement: S3 File — (PDF) [file pone.0246245.s005.pdf]

ClinicalTrials.gov PRS DRAFT Receipt (Working Version)
Last Update: 06/11/2019 21:58

ClinicalTrials.gov ID: NCT03950115

Study Identification

Unique Protocol ID: 2019-04-066

Brief Title: Effects and Prognostic Factors of Intensive Pulse Light Treatment for Meibomian Gland Dysfunction

Official Title: Effects and Prognostic Factors of Intensive Pulse Light Treatment for Meibomian Gland Dysfunction

Secondary IDs:

Study Status

Record Verification: June 2019

Overall Status: Active, not recruiting

Study Start: April 18, 2019 [Actual]

Primary Completion: June 18, 2019 [Anticipated]

Study Completion: July 18, 2019 [Anticipated]

Sponsor/Collaborators

Sponsor: Samsung Medical Center

Responsible Party: Principal Investigator
Investigator: tae-young chung [tychung]
Official Title: Professor
Affiliation: Samsung Medical Center

Collaborators:

Oversight

U.S. FDA-regulated Drug: No

U.S. FDA-regulated Device: No

U.S. FDA IND/IDE: No

Human Subjects Review: Board Status: Approved
Approval Number: SMC 2019-04-066
Board Name: Institutional Review Board of Samsung Medical Center
Board Affiliation: Samsung Medical Center, Seoul, Korea
Phone: 82-2-3410-2980
Email: hj0503.lee@samsung.com
Address:

50, Irwon-Dong, Gangnam-Gu, Seoul, Korea 135-710

Data Monitoring:

Study Description

Brief Summary: The investigators are going to Investigate the comparative efficacy of intense pulsed light therapy alone with that of intense pulsed light plus meibomian gland expression for meibomian gland dysfunction.

Detailed Description: Enrolled patients are going to be randomly assigned to two groups. All of the patients will undergo four treatment sessions in total, which are two weeks apart. Group 1 will undergo two sessions of intense pulsed light therapy with meibomian gland expression, as well as two sessions of intense pulsed light alone. Group 2 will receive two sessions of intense pulsed light therapy alone, and two sessions of intense pulsed light therapy with meibomian gland expression. The following parameters will be measured at baseline, 2 weeks after the second treatment session, and 2 weeks after the fourth treatment session: tearfilm break-up time, Oxford grade for corneal staining, meibomian gland expressibility, meibum quality, and ocular surface disease index.

Conditions

Conditions: Meibomian Gland Dysfunction

Keywords:

Study Design

Study Type: Interventional

Primary Purpose: Treatment

Study Phase: N/A

Interventional Study Model: Crossover Assignment

Number of Arms: 2

Masking: Single (Outcomes Assessor)

Allocation: Randomized

Enrollment: 80 [Anticipated]

Arms and Interventions

Arms	Assigned Interventions
Active Comparator: Group 1	Device: Intense Pulsed Light (IPL)<br>IPL therapy will be performed with the M22® (Lumenis, Dreieich, Germany). IPL treatment is going to be administered to the skin below the lower eyelid. Before treatment, the eyes will be protected with opaque goggles. Ultrasound gel is going to be applied to the patient's face from tragus to tragus including the nose in order to conduct the light, help to spread the energy evenly, and provide a degree of protection. The intensity of the IPL treatment will range from 9.8J/cm2 to 13J/cm2 according to Fitzpatrick Skin Type Grading.

Arms	Assigned Interventions
	Other Names:<br>• Meibomian gland expression
Active Comparator: Group 2	Device: Intense Pulsed Light (IPL)<br>IPL therapy will be performed with the M22® (Lumenis, Dreieich, Germany). IPL treatment is going to be administered to the skin below the lower eyelid. Before treatment, the eyes will be protected with opaque goggles. Ultrasound gel is going to be applied to the patient's face from tragus to tragus including the nose in order to conduct the light, help to spread the energy evenly, and provide a degree of protection. The intensity of the IPL treatment will range from 9.8J/cm2 to 13J/cm2 according to Fitzpatrick Skin Type Grading.<br>Other Names:<br>• Meibomian gland expression

Outcome Measures

Primary Outcome Measure:

1. Change from baseline tearfilm break-up time at 2 weeks after the last treatment session
   [Time Frame: Baseline and 2 weeks after the last treatment session]
2. Change from baseline Oxford grade for corneal staining at 2 weeks after the last treatment session
   Oxford grade for staining was assessed on a scale of 0 to 5. It was scaled according to the degree of corneal staining as follow: 0 (absent), 1 (minimal), 2 (mild), 3 (moderate), 4 (marked), and 5 (severe)

   [Time Frame: Baseline and 2 weeks after the last treatment session]
3. Change from baseline meibomian gland expressibility score at 2 weeks after the last treatment session
   The meibomian gland expressibility was assessed on a scale of 0 to 3 in five glands on the central lower lid. It was scaled according to the number of glands expressible, as follows: 0 (all glands), 1 (three to four glands), 2 (one to two glands) and 3 (no glands)

   [Time Frame: Baseline and 2 weeks after the last treatment session]
4. Change from baseline meibum quality score at 2 weeks after the last treatment session
   The meibum quality score were divided into the following four degrees: 0 (clear), 1 (cloudy), 2 (granular), and 3 (toothpaste)

   [Time Frame: Baseline and 2 weeks after the last treatment session]
5. Change from baseline ocular surface disease index at 2 weeks after the last treatment session
   The ocular surface diseases index score range from 0 to 100 based on the result of standardized ocular surface disease index questionnaire. Higher value represent worse subjective symptom.

   [Time Frame: Baseline and 2 weeks after the last treatment session]

Eligibility

Minimum Age:

Maximum Age:

Sex: All

Gender Based:

Accepts Healthy Volunteers: No

Criteria: Inclusion Criteria:

- Clinical diagnosis of meibomian gland dysfunction

Exclusion Criteria:

- Medical conditions in which IPL is contraindicated (pregnancy, breastfeeding, lupus, and any major uncontrolled health problem).
- Contact lens wearer
- Previous ocular surgery
- Previous thermal treatment for dry eye disease (e.g. LipiFlow)

Contacts/Locations

Central Contact Person: Tae-Young Chung, M.D., Ph.D.
Telephone: +82-10-9933-3563
Email: tychung@skku.edu

Central Contact Backup:

Study Officials:

Locations: **South Korea**
Samsung Medical Center
Seoul, South Korea
Contact: Tae-Young Chung +82-10-9933-3563 tychung@skku.edu

IPDSharing

Plan to Share IPD: No

References

Citations:

Links:

Available IPD/Information:
