# Supplementary material for: Intense pulsed light plus meibomian gland expression versus intense pulsed light alone for meibomian gland dysfunction: A randomized crossover study
Source: PLoS One. 2021 Mar 4;16(3):e0246245. doi: 10.1371/journal.pone.0246245 (PMC7932142; doi:10.1371/journal.pone.0246245)
Supplement: S4 File — (DOCX) [file pone.0246245.s006.docx]

**<Study Description>**

Brief Summary: The investigators are going to Investigate the comparative efficacy of intense pulsed light therapy alone with that of intense pulsed light plus meibomian gland expression for meibomian gland dysfunction.

Detailed Description: This is a prospective, non-significant risk, randomized multicenter cross-over study evaluating role of meibomian gland expression when it is combined with intense pulsed light for meibomian gland dysfunction. All subjects undergo examination to determine study eligibility and to capture the Baseline status prior to the treatment. Subjects will be randomly assigned to two groups. All of the patients will undergo four treatment sessions in total, which are two weeks apart. Group 1 will undergo two sessions of intense pulsed light therapy with meibomian gland expression, as well as two sessions of intense pulsed light alone. Group 2 will receive two sessions of intense pulsed light therapy alone, and two sessions of intense pulsed light therapy with meibomian gland expression. The following parameters will be measured at baseline, 2 weeks after the second treatment session, and 2 weeks after the fourth treatment session: tearfilm break-up time, Oxford grade for corneal staining, meibomian gland expressibility, meibum quality, and ocular surface disease index.

**<Study Design>**

Study type: Interventional (Clinical Trial)

Enrollment: 60 patients

Allocation: Randomized

Intervention Model: Crossover Assignment

Masking: Single (Outcomes Assessor)

Primary Purpose: Treatment

Official title: Effects and Prognostic Factors of Intensive Pulse Light Treatment for

Meibomian Gland Dysfunction

Study Start date: April 18, 2019

Actual primary Completion date: July 18, 2019

Actual study completion date: July 18, 2019

**<Arms and Interventions>**

All of patients underwent four treatment sessions that were two weeks apart. Group 1 underwent.

Group 1: Intense Pulsed Light (IPL) therapy with MGX at the first and second treatment sessions, and IPL therapy alone at the third and fourth treatment sessions

Group 2: IPL therapy alone at the first and second treatment sessions, and IPL therapy with MGX at the third and fourth treatment sessions

Device: Intense Pulsed Light (IPL)

IPL therapy will be performed with the M22® (Lumenis, Dreieich, Germany). IPL treatment is going to be administered to the skin below the lower eyelid. Before treatment, the eyes will be protected with opaque goggles. Ultrasound gel is going to be applied to the patient's face from tragus to tragus including the nose in order to conduct the light, help to spread the energy evenly, and provide a degree of protection. The intensity of the IPL treatment will range from 9.8J/cm2 to 13J/cm2 according to Fitzpatrick Skin Type Grading.

Procedure: manual meibomian gland expression

Meibomian gland expression will be performed immediately after IPL treatment on both upper and lower eyelids of each eye. In order to minimize pain during this procedure, the eye will be numbed with a solution of proparacaine HCl 0.5% (Alcaine; Alcon Laboratories, Fort Worth, TX). Meibomian gland expression will be then performed by squeezing the meibomian glands with meibomian gland expressor forceps, or with two Q-tips positioned on either side of the meibomian glands.

**<Outcome measures>**

Patients were evaluated immediately before the first treatment session (or the baseline), immediately before the third treatment session (first follow-up), and two weeks after the fourth treatment session (second follow-up). From baseline to second follow-up, each patient was treated and followed for a total of eight weeks.

**Primary outcome**

In order to separately evaluate the effect of meibomian gland expression on score improvement, the generalized estimating equation (GEE) method was employed. For the GEE model, dependent variables were defined as score changes (of tearfilm break-up time, Oxford grade for orneal staining, Meibomian gland expressibility, meibum quality, and ocular surface disease index) from baseline to the follow-up evaluations. First, univariable analysis was performed with age, sex, and baseline parameters as confounders. In addition, any parameters with a p-value <0.1 on univariable analysis were adjusted at the final GEE analysis model for meibomian gland expression effect.

Parameters

1. Tearfilm break-up time

The Investigator or designee measures tear break-up time (TBUT) under a slit-lamp biomicroscope following instillation of fluorescein dye in the eye using the Dry Eye Test (DET) method. Tear film break-up is defined as the first observed break-up of the tear film following the third blink. Using a stopwatch to record the time, start the stopwatch as soon as the subject opens his/her eyes after the third blink and stop the stopwatch when the first break-up of the tear film is observed. Any values recorded as greater than 20 seconds were converted to 20 seconds in the analysis. Three separate measurements were taken for each eye and were averaged for analysis. A higher tear break-up time indicates better tear film stability.

2. Oxford grade for corneal staining

Oxford grade for staining was assessed on a scale of 0 to 5. It was scaled according to the degree of corneal staining as follow: 0 (absent), 1 (minimal), 2 (mild), 3 (moderate), 4 (marked), and 5 (severe)

3. Meibomian gland expressibility score

Meibomian gland assessment was performed to evaluate the function of the meibomian glands based on the secretion characteristics from the gland orifices along the lower eyelid. Under a slit-lamp biomicrosope, the gland orifices were evaluated using a handheld instrument, Meibomian Gland Evaluator. A central 5 glands of the lower eyelid were evaluated. The meibomian gland expressibility was assessed on a scale of 0 to 3. It was scaled according to the number of glands expressible, as follows: 0 (all glands), 1 (three to four glands), 2 (one to two

glands) and 3 (no glands)

4. Meibum quality score

The meibum quality score were divided into the following four degrees: 0 (clear), 1 (cloudy), 2 (granular), and 3 (toothpaste)

5. Change in Dry Eye Questionnaire Score

Ocular surface disease index (OSDI) is a 12-item questionnaire designed to provide a rapid assessment of the symptoms of ocular irritation consistent with dry eye disease and their impact on vision-related functioning. Dry eye symptoms assessed using the OSDI questionnaire are sensitivity to light, grittiness, pain or soreness, blurred vision and poor vision. The assessment considers the frequency that problems with the eyes limit performance in reading, driving at night, working with a computer or bank machine, and watching television. Also, the frequency that eyes feel uncomfortable is assessed in windy conditions, areas with low humidity, and air-conditioned areas. The frequency scale is: 0 (none of the time), 1 (some of the time), 2 (half of the time), 3 (most of the time) and 4 (all of the time). The subject can answer not applicable (N/A) if the subject did not experience the situation or condition in the past week. Total OSDI score is calculated as the sum of frequency scores for all symptoms multiplied by 25 and divided by the number of questions answered with a range from 0 to 100. A lower OSDI score represents less disability from dry eye symptoms.

**Secondary outcomes**

1. The clinical parameters of both groups were compared at each point of the evaluation (baseline, first follow-up, second follow-up) using the independent t-test.

2. Analysis of the improvement after therapy was performed using paired 𝑡-tests, which compared the BUT, Oxford grade, MGE, MQ, and OSDI scores at baseline, first follow-up, and second follow-up

**<Eligibility Criteria>**

Ages Eligible for Study: All

Sexes Eligible for Study: All

Accepts Healthy Volunteers: No

**Criteria**

Inclusion Criteria:

-Must be able to comply with protocol including study randomization; completion of planned bilateral cataract surgery; no use of other MGD or dry eye treatments during the study; and attendance at all study visits

-Meibomian gland dysfunction in both eyes

Exclusion Criteria:

-Systemic disease conditions that causes dry eye

-Use of systemic medications known to cause dry eye

-Use of other MGD or dry eye treatments except for over the counter lubricants or dietary supplements

-History of any of the following ocular conditions in the past 3 months: surgery, trauma, Herpes infection, recurrent inflammation, punctal plug insertion or punctal occlusion

-Presence of any medical condition which IPL is contraindicated (e.g. including pregnancy, breastfeeding, lupus, and any major uncontrolled health problem)

-Presence of any of the following active conditions: ocular infection, ocular inflammation, moderate to severe allergic conjunctivitis, severe eyelid inflammation, eyelid abnormality that affects lid function, or ocular surface abnormality that compromises corneal integrity

-Participation in another ophthalmic drug or device trial in the past month
